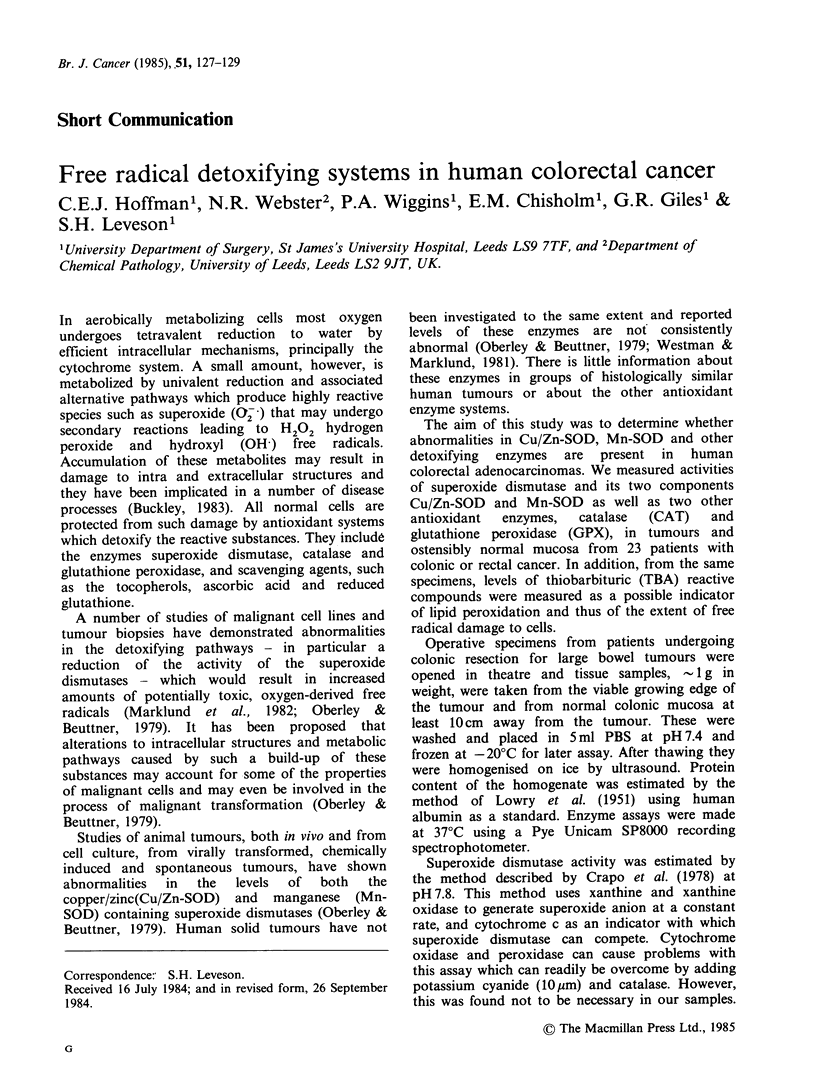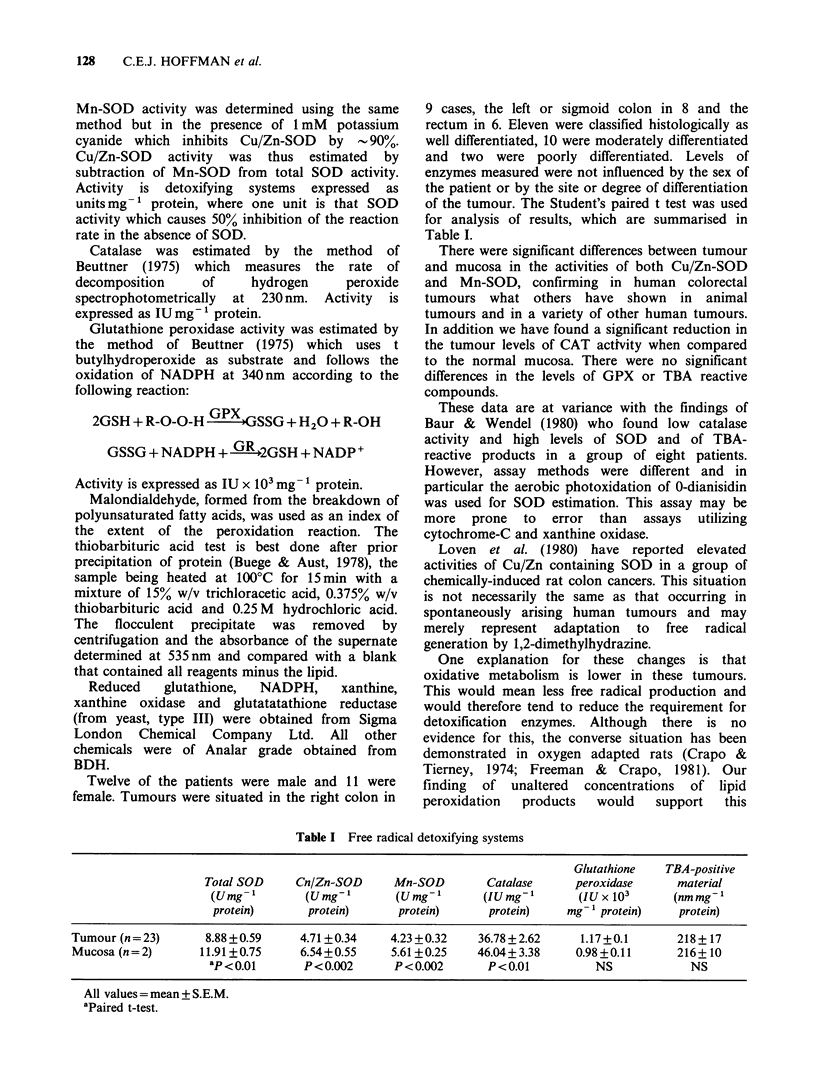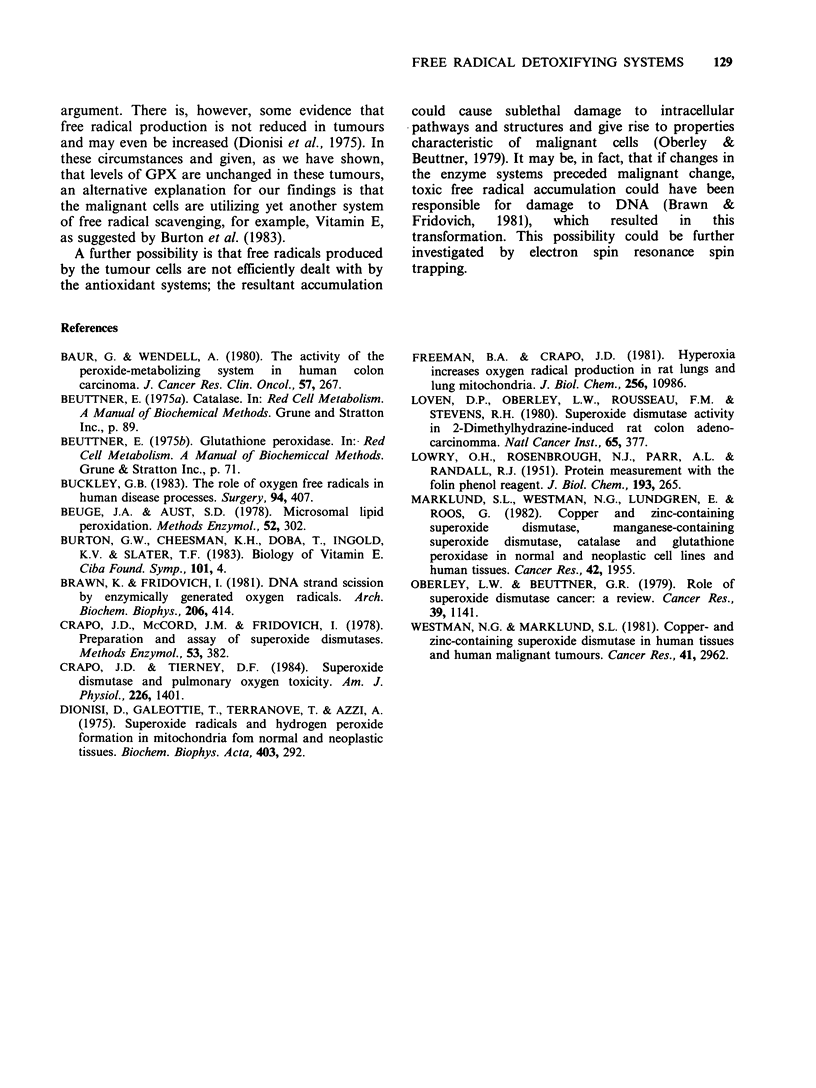# Free radical detoxifying systems in human colorectal cancer.

**DOI:** 10.1038/bjc.1985.18

**Published:** 1985-01

**Authors:** C. E. Hoffman, N. R. Webster, P. A. Wiggins, E. M. Chisholm, G. R. Giles, S. H. Leveson


Br. J. Cancer (1985), 51, 127–129

### Short Communication

### Free radical detoxifying systems in human colorectal cancer

C.E.J. Hoffman[1], N.R. Webster[2], P.A. Wiggins[1], E.M. Chisholm[1], G.R. Giles[1] & S.H. Leveson[1]

[1]*University Department of Surgery, St James's University Hospital, Leeds LS9 7TF, and* [2]*Department of Chemical Pathology, University of Leeds, Leeds LS2 9JT, UK.*

In aerobically metabolizing cells most oxygen undergoes tetravalent reduction to water by efficient intracellular mechanisms, principally the cytochrome system. A small amount, however, is metabolized by univalent reduction and associated alternative pathways which produce highly reactive species such as superoxide ($O_2^{-}\cdot$) that may undergo secondary reactions leading to $H_2O_2$ hydrogen peroxide and hydroxyl ($OH\cdot$) free radicals. Accumulation of these metabolites may result in damage to intra and extracellular structures and they have been implicated in a number of disease processes (Buckley, 1983). All normal cells are protected from such damage by antioxidant systems which detoxify the reactive substances. They include the enzymes superoxide dismutase, catalase and glutathione peroxidase, and scavenging agents, such as the tocopherols, ascorbic acid and reduced glutathione.

A number of studies of malignant cell lines and tumour biopsies have demonstrated abnormalities in the detoxifying pathways – in particular a reduction of the activity of the superoxide dismutases – which would result in increased amounts of potentially toxic, oxygen-derived free radicals (Marklund *et al.*, 1982; Oberley & Beuttner, 1979). It has been proposed that alterations to intracellular structures and metabolic pathways caused by such a build-up of these substances may account for some of the properties of malignant cells and may even be involved in the process of malignant transformation (Oberley & Beuttner, 1979).

Studies of animal tumours, both *in vivo* and from cell culture, from virally transformed, chemically induced and spontaneous tumours, have shown abnormalities in the levels of both the copper/zinc(Cu/Zn-SOD) and manganese (Mn-SOD) containing superoxide dismutases (Oberley & Beuttner, 1979). Human solid tumours have not been investigated to the same extent and reported levels of these enzymes are not consistently abnormal (Oberley & Beuttner, 1979; Westman & Marklund, 1981). There is little information about these enzymes in groups of histologically similar human tumours or about the other antioxidant enzyme systems.

The aim of this study was to determine whether abnormalities in Cu/Zn-SOD, Mn-SOD and other detoxifying enzymes are present in human colorectal adenocarcinomas. We measured activities of superoxide dismutase and its two components Cu/Zn-SOD and Mn-SOD as well as two other antioxidant enzymes, catalase (CAT) and glutathione peroxidase (GPX), in tumours and ostensibly normal mucosa from 23 patients with colonic or rectal cancer. In addition, from the same specimens, levels of thiobarbituric (TBA) reactive compounds were measured as a possible indicator of lipid peroxidation and thus of the extent of free radical damage to cells.

Operative specimens from patients undergoing colonic resection for large bowel tumours were opened in theatre and tissue samples, ~1 g in weight, were taken from the viable growing edge of the tumour and from normal colonic mucosa at least 10 cm away from the tumour. These were washed and placed in 5 ml PBS at pH 7.4 and frozen at −20°C for later assay. After thawing they were homogenised on ice by ultrasound. Protein content of the homogenate was estimated by the method of Lowry *et al.* (1951) using human albumin as a standard. Enzyme assays were made at 37°C using a Pye Unicam SP8000 recording spectrophotometer.

Superoxide dismutase activity was estimated by the method described by Crapo *et al.* (1978) at pH 7.8. This method uses xanthine and xanthine oxidase to generate superoxide anion at a constant rate, and cytochrome c as an indicator with which superoxide dismutase can compete. Cytochrome oxidase and peroxidase can cause problems with this assay which can readily be overcome by adding potassium cyanide (10 $\mu$m) and catalase. However, this was found not to be necessary in our samples.

Correspondence: S.H. Leveson.
Received 16 July 1984; and in revised form, 26 September 1984.

© The Macmillan Press Ltd., 1985

Mn-SOD activity was determined using the same method but in the presence of 1 mM potassium cyanide which inhibits Cu/Zn-SOD by ~90%. Cu/Zn-SOD activity was thus estimated by subtraction of Mn-SOD from total SOD activity. Activity is detoxifying systems expressed as units $mg^{-1}$ protein, where one unit is that SOD activity which causes 50% inhibition of the reaction rate in the absence of SOD.

Catalase was estimated by the method of Beuttner (1975) which measures the rate of decomposition of hydrogen peroxide spectrophotometrically at 230 nm. Activity is expressed as IU $mg^{-1}$ protein.

Glutathione peroxidase activity was estimated by the method of Beuttner (1975) which uses t butylhydroperoxide as substrate and follows the oxidation of NADPH at 340 nm according to the following reaction:

$$2GSH + R\text{-}O\text{-}O\text{-}H \xrightarrow{GPX} GSSG + H_2O + R\text{-}OH$$

$$GSSG + NADPH + \xrightarrow{GR} 2GSH + NADP^+$$

Activity is expressed as $IU \times 10^3 mg^{-1}$ protein.

Malondialdehyde, formed from the breakdown of polyunsaturated fatty acids, was used as an index of the extent of the peroxidation reaction. The thiobarbituric acid test is best done after prior precipitation of protein (Buege & Aust, 1978), the sample being heated at 100°C for 15 min with a mixture of 15% w/v trichloracetic acid, 0.375% w/v thiobarbituric acid and 0.25 M hydrochloric acid. The flocculent precipitate was removed by centrifugation and the absorbance of the supernate determined at 535 nm and compared with a blank that contained all reagents minus the lipid.

Reduced glutathione, NADPH, xanthine, xanthine oxidase and glutatatathione reductase (from yeast, type III) were obtained from Sigma London Chemical Company Ltd. All other chemicals were of Analar grade obtained from BDH.

Twelve of the patients were male and 11 were female. Tumours were situated in the right colon in 9 cases, the left or sigmoid colon in 8 and the rectum in 6. Eleven were classified histologically as well differentiated, 10 were moderately differentiated and two were poorly differentiated. Levels of enzymes measured were not influenced by the sex of the patient or by the site or degree of differentiation of the tumour. The Student's paired t test was used for analysis of results, which are summarised in Table I.

There were significant differences between tumour and mucosa in the activities of both Cu/Zn-SOD and Mn-SOD, confirming in human colorectal tumours what others have shown in animal tumours and in a variety of other human tumours. In addition we have found a significant reduction in the tumour levels of CAT actfvity when compared to the normal mucosa. There were no significant differences in the levels of GPX or TBA reactive compounds.

These data are at variance with the findings of Baur & Wendel (1980) who found low catalase activity and high levels of SOD and of TBA-reactive products in a group of eight patients. However, assay methods were different and in particular the aerobic photoxidation of 0-dianisidin was used for SOD estimation. This assay may be more prone to error than assays utilizing cytochrome-C and xanthine oxidase.

Loven *et al.* (1980) have reported elevated activities of Cu/Zn containing SOD in a group of chemically-induced rat colon cancers. This situation is not necessarily the same as that occurring in spontaneously arising human tumours and may merely represent adaptation to free radical generation by 1,2-dimethylhydrazine.

One explanation for these changes is that oxidative metabolism is lower in these tumours. This would mean less free radical production and would therefore tend to reduce the requirement for detoxification enzymes. Although there is no evidence for this, the converse situation has been demonstrated in oxygen adapted rats (Crapo & Tierney, 1974; Freeman & Crapo, 1981). Our finding of unaltered concentrations of lipid peroxidation products would support this

**Table I** Free radical detoxifying systems

| | *Total SOD ($U mg^{-1}$ protein)* | *Cn/Zn-SOD ($U mg^{-1}$ protein)* | *Mn-SOD ($U mg^{-1}$ protein)* | *Catalase ($IU mg^{-1}$ protein)* | *Glutathione peroxidase ($IU \times 10^3$ $mg^{-1}$ protein)* | *TBA-positive material ($nm mg^{-1}$ protein)* |
|---|---|---|---|---|---|---|
| Tumour ($n=23$) | 8.88±0.59 | 4.71±0.34 | 4.23±0.32 | 36.78±2.62 | 1.17±0.1 | 218±17 |
| Mucosa ($n=2$) | 11.91±0.75 | 6.54±0.55 | 5.61±0.25 | 46.04±3.38 | 0.98±0.11 | 216±10 |
| | $^a P<0.01$ | $P<0.002$ | $P<0.002$ | $P<0.01$ | NS | NS |

All values = mean ± S.E.M.
[a]Paired t-test.

argument. There is, however, some evidence that free radical production is not reduced in tumours and may even be increased (Dionisi *et al.*, 1975). In these circumstances and given, as we have shown, that levels of GPX are unchanged in these tumours, an alternative explanation for our findings is that the malignant cells are utilizing yet another system of free radical scavenging, for example, Vitamin E, as suggested by Burton *et al.* (1983).

A further possibility is that free radicals produced by the tumour cells are not efficiently dealt with by the antioxidant systems; the resultant accumulation could cause sublethal damage to intracellular pathways and structures and give rise to properties characteristic of malignant cells (Oberley & Beuttner, 1979). It may be, in fact, that if changes in the enzyme systems preceded malignant change, toxic free radical accumulation could have been responsible for damage to DNA (Brawn & Fridovich, 1981), which resulted in this transformation. This possibility could be further investigated by electron spin resonance spin trapping.

### References

BAUR, G. & WENDELL, A. (1980). The activity of the peroxide-metabolizing system in human colon carcinoma. *J. Cancer Res. Clin. Oncol.*, **57**, 267.

BEUTTNER, E. (1975*a*). Catalase. In: *Red Cell Metabolism. A Manual of Biochemical Methods*. Grune and Stratton Inc., p. 89.

BEUTTNER, E. (1975*b*). Glutathione peroxidase. In:· *Red Cell Metabolism. A Manual of Biochemiccal Methods*. Grune & Stratton Inc., p. 71.

BUCKLEY, G.B. (1983). The role of oxygen free radicals in human disease processes. *Surgery*, **94**, 407.

BEUGE, J.A. & AUST, S.D. (1978). Microsomal lipid peroxidation. *Methods Enzymol.*, **52**, 302.

BURTON, G.W., CHEESMAN, K.H., DOBA, T., INGOLD, K.V. & SLATER, T.F. (1983). Biology of Vitamin E. *Ciba Found. Symp.*, **101**, 4.

BRAWN, K. & FRIDOVICH, I. (1981). DNA strand scission by enzymically generated oxygen radicals. *Arch. Biochem. Biophys.*, **206**, 414.

CRAPO, J.D., McCORD, J.M. & FRIDOVICH, I. (1978). Preparation and assay of superoxide dismutases. *Methods Enzymol.*, **53**, 382.

CRAPO, J.D. & TIERNEY, D.F. (1984). Superoxide dismutase and pulmonary oxygen toxicity. *Am. J. Physiol.*, **226**, 1401.

DIONISI, D., GALEOTTIE, T., TERRANOVE, T. & AZZI, A. (1975). Superoxide radicals and hydrogen peroxide formation in mitochondria fom normal and neoplastic tissues. *Biochem. Biophys. Acta*, **403**, 292.

FREEMAN, B.A. & CRAPO, J.D. (1981). Hyperoxia increases oxygen radical production in rat lungs and lung mitochondria. *J. Biol. Chem.*, **256**, 10986.

LOVEN, D.P., OBERLEY, L.W., ROUSSEAU, F.M. & STEVENS, R.H. (1980). Superoxide dismutase activity in 2-Dimethylhydrazine-induced rat colon adenocarcinomma. *Natl Cancer Inst.*, **65**, 377.

LOWRY, O.H., ROSENBROUGH, N.J., PARR, A.L. & RANDALL, R.J. (1951). Protein measurement with the folin phenol reagent. *J. Biol. Chem.*, **193**, 265.

MARKLUND, S.L., WESTMAN, N.G., LUNDGREN, E. & ROOS, G. (1982). Copper and zinc-containing superoxide dismutase, manganese-containing superoxide dismutase, catalase and glutathione peroxidase in normal and neoplastic cell lines and human tissues. *Cancer Res.*, **42**, 1955.

OBERLEY, L.W. & BEUTTNER, G.R. (1979). Role of superoxide dismutase cancer: a review. *Cancer Res.*, **39**, 1141.

WESTMAN, N.G. & MARKLUND, S.L. (1981). Copper- and zinc-containing superoxide dismutase in human tissues and human malignant tumours. *Cancer Res.*, **41**, 2962.